# Identification of plasma lipid species as promising diagnostic markers for prostate cancer

**DOI:** 10.1186/s12911-020-01242-7

**Published:** 2020-09-24

**Authors:** Xiaoli Chen, Yong Zhu, Mayumi Jijiwa, Masaki Nasu, Junmei Ai, Shengming Dai, Bin Jiang, Jicai Zhang, Gang Huang, Youping Deng

**Affiliations:** 1 Medical Science Laboratory, the Fourth Affiliated Hospital of Guangxi Medical University, Liuzhou, Guangxi China; 2 Department of Medicine, Rush University Medical Center, Chicago, IL USA; 3 National Medical Centre of Colorectal Disease, The Third Affiliated Hospital of Nanjing University of Chinese Medicine, Nanjing, P. R. China; 4 Bioinformatics Core, Department of Quantitative Health Sciences, University of Hawaii John A. Burns School of Medicine, Honolulu, HI USA; 5 Department of Laboratory Medicine, Shiyan Taihe Hospital, College of Biomedical Engineering, Hubei University of Medicine, Shiyan, Hubei 442000 P. R. China; 6 Shanghai Key Laboratory for Molecular Imaging, Shanghai University of Medicine and Health Sciences, Shanghai, 201318 P. R. China

**Keywords:** Lipidomics, Prostate cancer, Diagnosis, Metabolic pathway, LC-ESI-MS/MS

## Abstract

**Background:**

Prostate cancer is a very common and highly fatal in men. Current non-invasive detection methods like serum biomarker are unsatisfactory. Biomarkers with high accuracy for diagnostic of prostate cancer are urgently needed. Many lipid species have been found related to various cancers. The purpose of our study is to explore the diagnostic value of lipids for prostate cancer.

**Results:**

Using triple quadruple liquid chromatography electrospray ionization tandem mass spectrometry, we performed lipidomics profiling of 367 lipids on a total 114 plasma samples from 30 patients with prostate cancer, 38 patients with benign prostatic hyperplasia (BPH), and 46 male healthy controls to evaluate the lipids as potential biomarkers in the diagnosis of prostate cancer. Kyoto Encyclopedia of Genes and Genomes (KEGG) pathway database was used to construct the potential mechanism pathway. After statistical analysis, five lipids were identified as a panel of potential biomarkers for the detection of prostate cancer between prostate cancer group and the BPH group; the sensitivity, specificity, and area under curve (AUC) of the combination of these five lipids were 73.3, 81.6%, and 0.800, respectively. We also identified another panel of five lipids in distinguishing between prostate cancer group and the control group with predictive values of sensitivity at 76.7%, specificity at 80.4%, and AUC at 0.836, respectively. The glycerophospholipid metabolism pathway of the selected lipids was considered as the target pathway.

**Conclusions:**

Our study indicated that the identified plasma lipid biomarkers have potential in the diagnosis of prostate cancer.

## Background

Prostate cancer is the most frequently diagnosed cancer in men and the second leading cause of cancer-related death among men in the United States [1, 2]. Like other cancers, early detection is the key to successful treatment for prostate cancer. The serum/plasma biomarkers have the advantages of being noninvasive and highly reproducible at low costs. Thus, the diagnostic value of serum/plasma biomarkers in various cancers is a hotspot in recent research. The concentration of serum prostate-specific-antigen (PSA) is widely used in prostate cancer screening. However, the performance of serum PSA for the screening of prostate cancer is not satisfactory. The lack of specificity of PSA screening for diagnosing prostate cancer leads to a large number of false positive results. Many patients are subjected to unnecessary prostate biopsies which increase health-care costs [3]. Moreover, many patients with advanced prostate cancer often have normal PSA levels in clinical trials [4]. That makes patients miss the best time for treatment and thus subsequently results in poor prognosis. Therefore, new serum or plasma biomarkers with high accuracy are urgently needed.

Lipids, as a vital component of human biology, are involved in regulating many physiological activities, such as energy storage, structure, apoptosis, and signaling [5]. Many studies have been reported that the dysregulation of lipid metabolism was associated with various diseases [6–9]. Therefore, lipids and their metabolites can be considered as indicators to distinguish between health and disease. Lipidomics was proposed as one of the important research fields of metabolomics in 2003 [10]. Its research interest mainly focuses on the relative changes between composition and concentration of lipids in cells and in biological fluids [11], which can play an important diagnostic role in various cancers, such as ovarian cancer [12], kidney cancer [13], esophageal squamous cell carcinoma [14] and lung cancer [15, 16]. Furthermore, many studies have shown the correlation between dyslipidemia and BPH or prostate cancer [17–20]. Therefore, detection and evaluation of the lipid species in patients with prostate cancer are the hotspots in current researches.

In our study, we evaluated the lipid species in plasma taken from healthy people as control and patients with BPH or prostate cancer. As far as we know, this is the first comprehensive evaluation of plasma lipid profiles for BPH patients. Based on the comparison between prostate cancer and BPH or healthy control, we could provide more detailed classification of lipid biomarkers. The predominant metabolic map (map 00564) of all the selected lipid species was a glycerophospholipid pathway by Kyoto Encyclopedia of Genes and Genomes (KEGG). Our data indicated that the lipid species could be used as potential biomarkers in the diagnosis of prostate cancer.

## Results

### Characteristics of subjects

Characteristics of subjects are summarized in Table 1. Our study contained 114 subjects, composed of 30 patients with prostate cancer, 38 patients with BPH, and 46 healthy controls. The average age of prostate cancer group was 62.3 ± 5.5, BPH group was 64.7 ± 5.5, and healthy control group was 63.70 ± 6.28, respectively. The prostate cancer group had 23 (76.7%) Caucasian and 7 (23.3%) African American. In the BPH group, there were 30 (78.9%) Caucasian and 8 (21.1%) African American. Among the control group, there were 36 (78.3%) Caucasian and 10 (21.7%) African American. There was no significant age or racial bias among the three groups (^a^*P*, ^b^*P* > 0.05). Most of the prostate cancer patients had Gleason score of 6 or 7 (83.3%) (Gleason score of 6 in 14 patients, 7 in 11 patients, and above 8 in 5 cases).

**Table 1:** Characteristics of subjects

	Cancer	BPH	Control	^**a**^***P***-value	^**b**^***P***-value
**Age range (years, mean ± SD)**	62.3 ± 5.5	64.7 ± 5.5	63.7 ± 6.3	0.490	0.175
**Race**					
**Caucasian**	23	30	36	0.822	0.871
**African American**	7	8	10		
**Gleason Score**					
**6**	14	–	–	–	–
**7**	11	–	–	–	–
**8–10**	5	–	–	–	–

*BPH* benign prostatic hyperplasia, *SD* standard deviation, ^a^*P*-value for cancer vs. BPH, ^b^*P*-value for cancer vs. control

### Profiling of lipid species

**Fig. 1:**
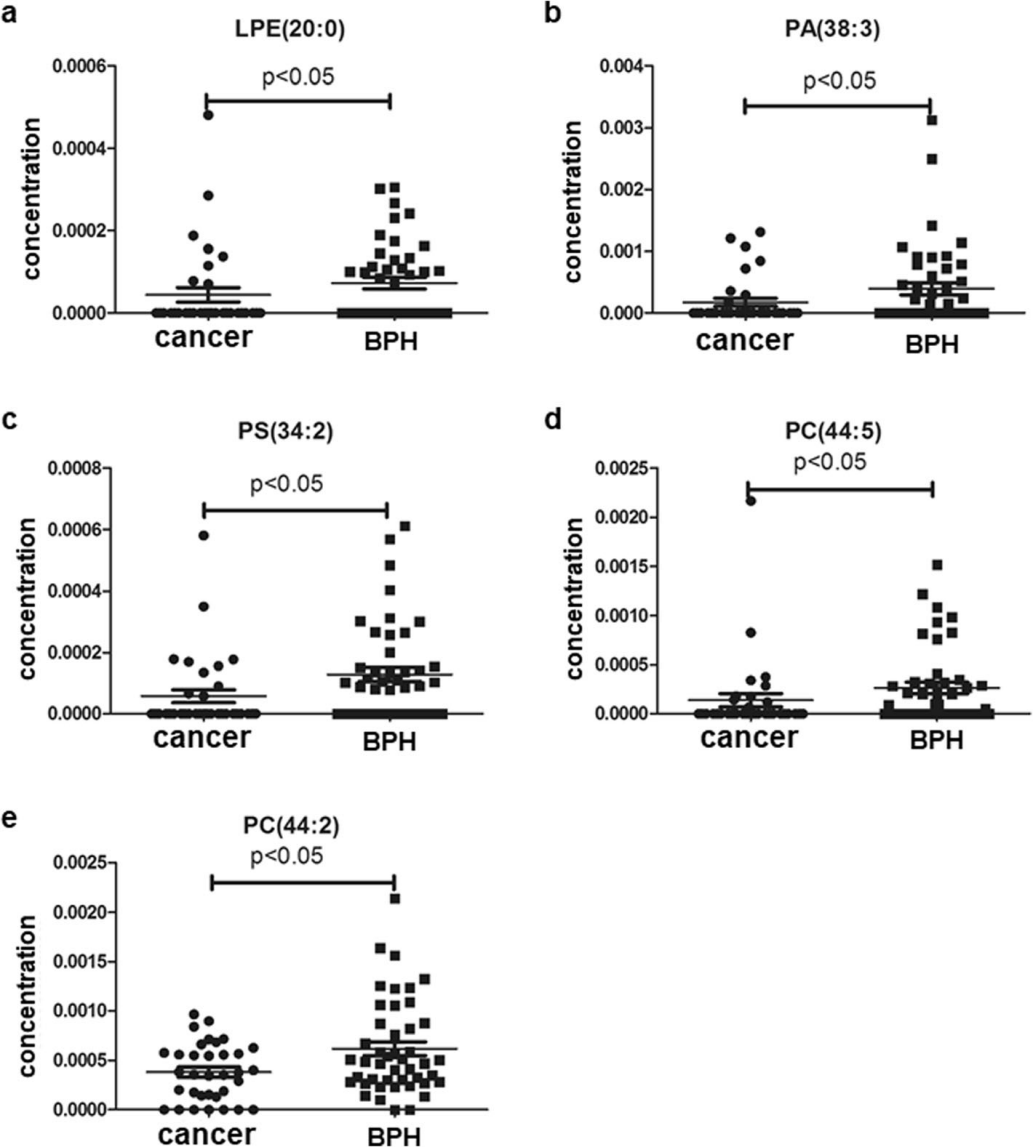
Plasma concentrations of 5 lipid species in the diagnosis of prostate cancer for prostate cancer group versus the BPH group

**Fig. 2:**
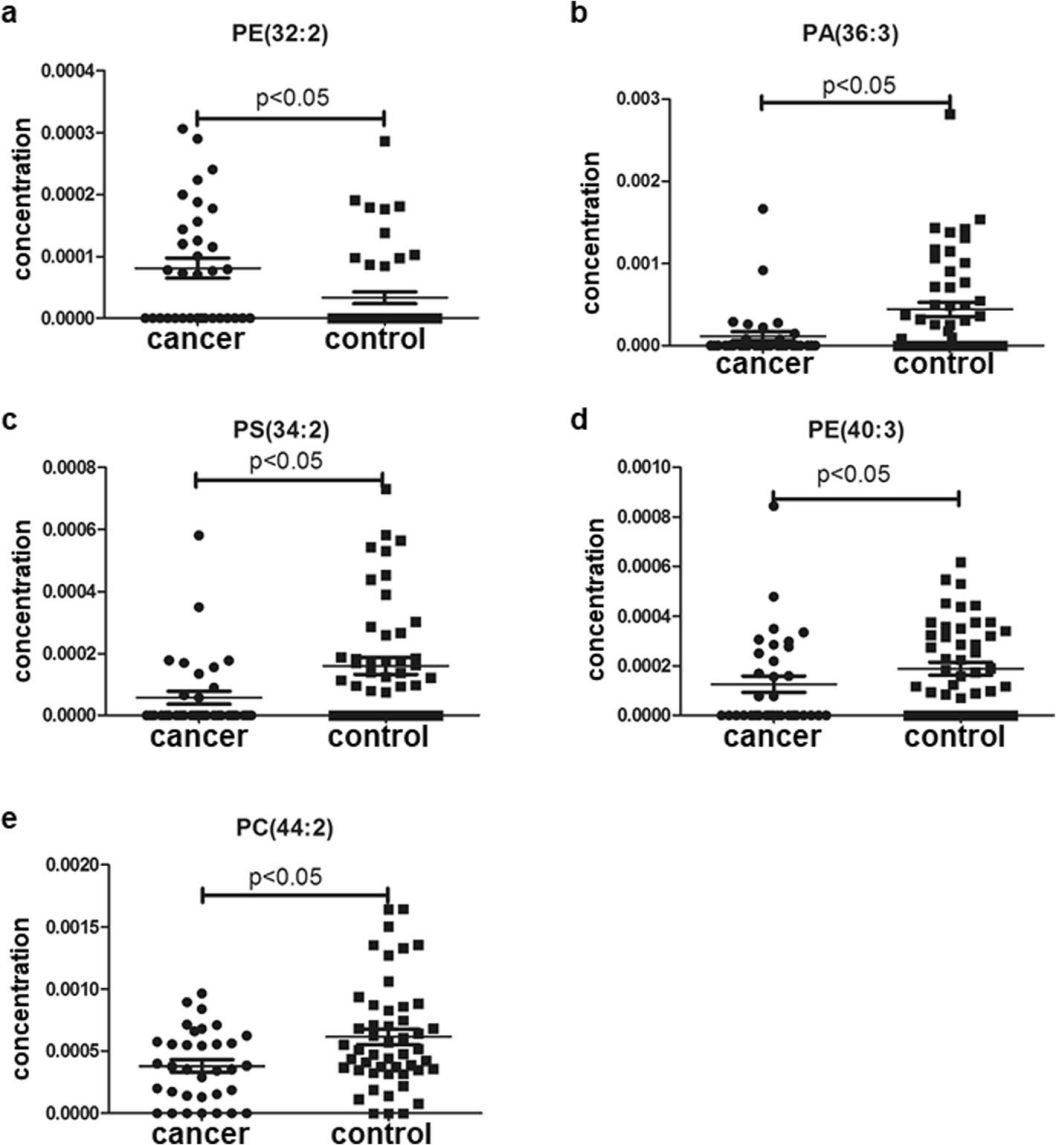
Plasma concentrations of 5 lipid species in the diagnosis of prostate cancer for prostate cancer group versus the male control group

**Fig. 3:**
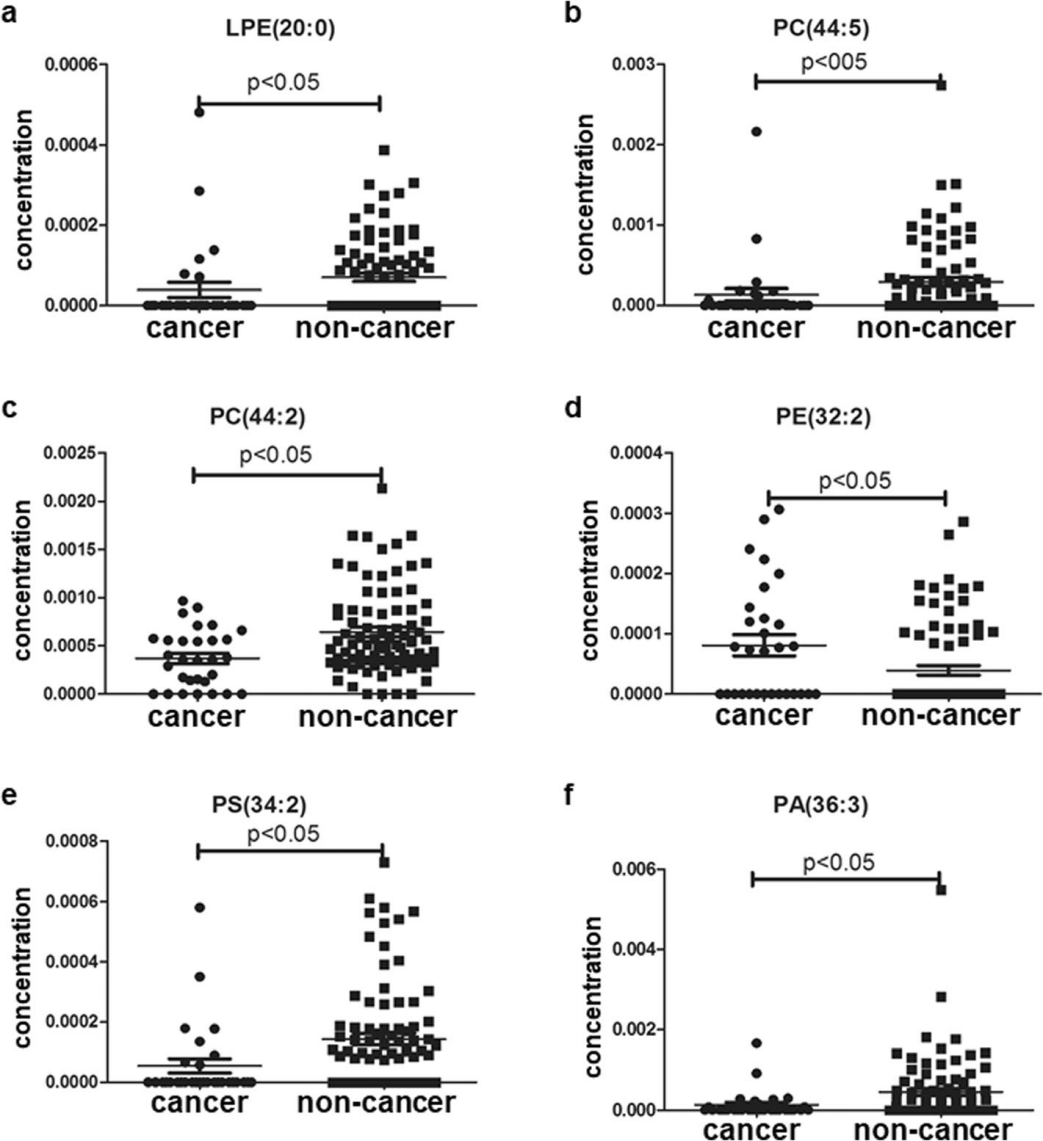
Plasma concentrations of 5 lipid species in the diagnosis of prostate cancer for prostate cancer group versus the non-cancer group (benign prostatic hyperplasia plus healthy controls)

Plasma lipid profiles, including 367 lipid species from 14 classes of phospholipids and 1 class of cholesterol ester, were identified in all subjects by lipidomics. The concentrations of lipid species in all subjects were analyzed. In the prostate cancer versus BPH group, the most significant difference of mean plasma concentration was seen in PC (44:2) (Fig. 1e, *p* = 0.004). The significant fold change was seen in PA (38:3) (Table 2, fold-change = − 2.34). In the prostate cancer versus healthy control group, the most significant difference in mean plasma concentration was seen in PS (34:2) (Fig. 2c, *p* = 0.002). The significant fold change was PA (36:3) (Table 2, fold-change = − 3.60). In the prostate cancer versus the non-cancer group (BPH group + control group), the most significant difference in mean plasma concentration was seen in PC (44:2) (Fig. 3c, *p* < 0.001). The significant fold change was PA (36:3) (Table 2, fold-change = − 3.45). These results indicated that lipid species could be served as biomarkers for the diagnosis of prostate cancer.

**Table 2:** The detection of lipid species as potential biomarkers for diagnosis of prostate cancer

Group	Lipid species	***P*** value	Fold-change	Sensitivity	Specificity	PPV	NPV	ROC Area
**Cancer vs. BPH**	**LPE (20:0)**	0.034	− 1.81	80.0%	44.7%	53.3%	73.9%	0.543
	**PA (38:3)**	0.018	−2.34	73.3%	55.3%	56.4%	72.4%	0.643
	**PS (34:2)**	0.009	−2.26	80.0%	57.9%	60%	78.6%	0.689
	**PC (44:5)**	0.014	−2.21	83.3%	50.0%	56.8%	79.2%	0.624
	**PC (44:2)**	0.004	−1.79	43.3%	89.5%	76.5%	66.7%	0.686
	**Combination of 5 lipids**	–	–	73.3%	81.6%	75.9%	79.5%	0.800
**Cancer vs. Control**	**PE (32:2)**	0.009	2.29	43.3%	84.8%	65%	69.6%	0.653
	**PA (36:3)**	0.007	−3.60	86.7%	47.8%	52%	84.6%	0.672
	**PS (34:2)**	0.002	2.91	80%	60.9%	57.1%	82.4%	0.704
	**PE (40:3)**	0.042	−1.43	23.3%	89.1%	58.3%	64.1%	0.628
	**PC (44:2)**	0.017	−1.72	40%	87%	66.7%	69.0%	0.661
	**Combination of 5 lipids**	–	–	76.7%	80.4%	71.9%	84.1%	0.836
**Cancer vs. Non-cancer**	**PA (36:3)**	0.011	−3.45	76.7%	44%	32.9%	84.1%	0.646
	**PS (34:2)**	0.001	−2.62	76.7%	59.5%	40.4%	87.7%	0.633
	**PE (32:2)**	0.010	2.04	30%	92.9%	60%	78.8%	0.614
	**LPE (20:0)**	0.021	−1.79	70.0%	41.7%	30.0%	79.5%	0.578
	**PC (44:5)**	0.021	−2.19	86.7%	39.3%	33.8%	89.2%	0.613
	**PC (44:2)**	0.001	−1.75	3.3%	94%	16.7%	78.8%	0.661
	**Combination of 6 lipids**	–	–	73.3%	82.1%	59.5%	89.6%	0.837

*BPH* benign prostatic hyperplasia, *PPV* positive predictive value, *NPV* negative predictive value, *ROC* receiver operating characteristic curve

### Identification of lipid species as biomarkers

According to the inclusion criteria described in Materials and methods section, five lipid species were selected as potential biomarkers for diagnosis of prostate cancer to distinguish prostate cancer from BPH, and another five lipid species to distinguish prostate cancer from healthy control. Six lipid species were selected as potential biomarkers that can detect prostate cancer out of non-cancer lesion. However, we did not observe significant difference of any lipid level between the BPH group and the control group (Data not shown). The concentration distribution of these selected lipid species was shown in Figs. 1, 2 and 3.

**Fig. 4:**
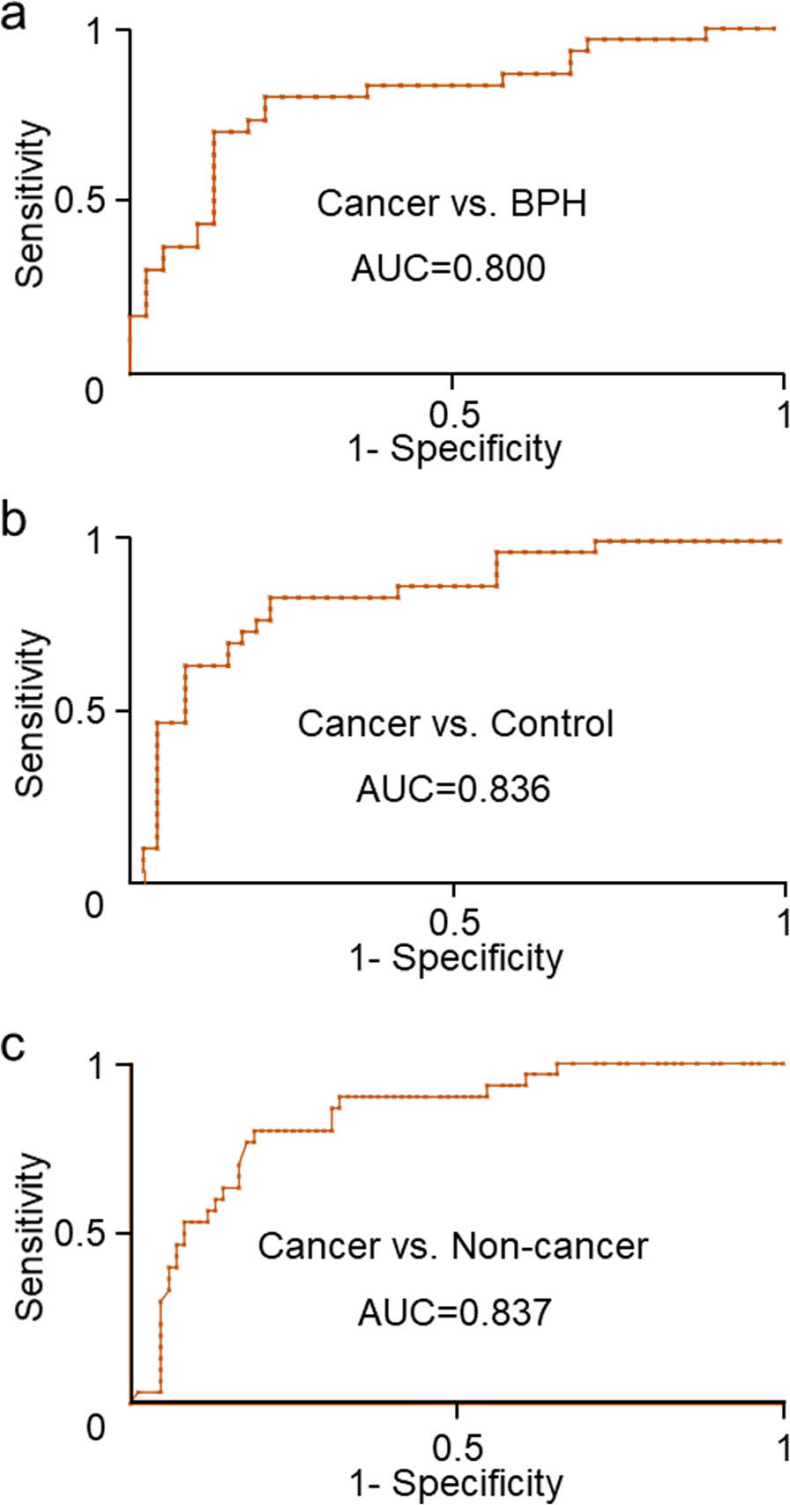
ROC curve of the combination of lipid species in prediction of prostate cancer. a. Prostate cancer versus benign prostatic hyperplasia. b. Prostate cancer versus healthy controls. c. prostate cancer versus non-cancer group (benign prostatic hyperplasia plus healthy controls)

Using the Weka 3.6 software, we could conduct predictive model to predict the diagnostic efficiency for the selected lipid species. As shown in Table 2, the diagnostic efficiency of the single lipid was not satisfactory. However, it might be significantly improved by the combination of the selected lipid species. From the results, we could find that the six selected lipids as potential markers to distinguish prostate cancer from non-cancer group (PA, PS, PE, LPE, PC (44:5) and PC (44:2)). Four out of those six lipids overlapped with prostate cancer vs. BPH or control group. According to the predictive model conducted by the Bagging classification algorithm and 10-fold cross validation between prostate cancer group and the BPH group, the algorithm correctly classified 53 out of 68 cases (a correct classification rate of 77.9%). The sensitivity, specificity, and AUC of five lipids-combined species in prostate cancer patients related to the BPH group were 73.3, 81.6%, and 0.800 (Table 2, Fig. 4a), respectively. For discriminating prostate cancer group from healthy control group, we correctly classified an overall 60 out of 76 patients (a correct classification rate of 78.9%), and five lipids-combined species showed a sensitivity of 76.7%, specificity of 80.4% and AUC of 0.836 (Table 2, Fig. 4b). According to the predictive model conducted by ADTree classification algorithm and 10-fold cross validation between prostate cancer and non-cancer group, the algorithm correctly clarified 91 out of the 114 cases (a correct classification rate of 79.8%). The sensitivity, specificity, and the AUC of these six lipid species in prostate cancer patients related to the non-cancer group were 73.3, 82.1%, and 0.837 (Table 2, Fig. 4c), respectively. These results indicated that the selected lipid species had certain value in the diagnosis of prostate cancer when combined.

### Selected lipids and pathway analysis

**Fig. 5:**
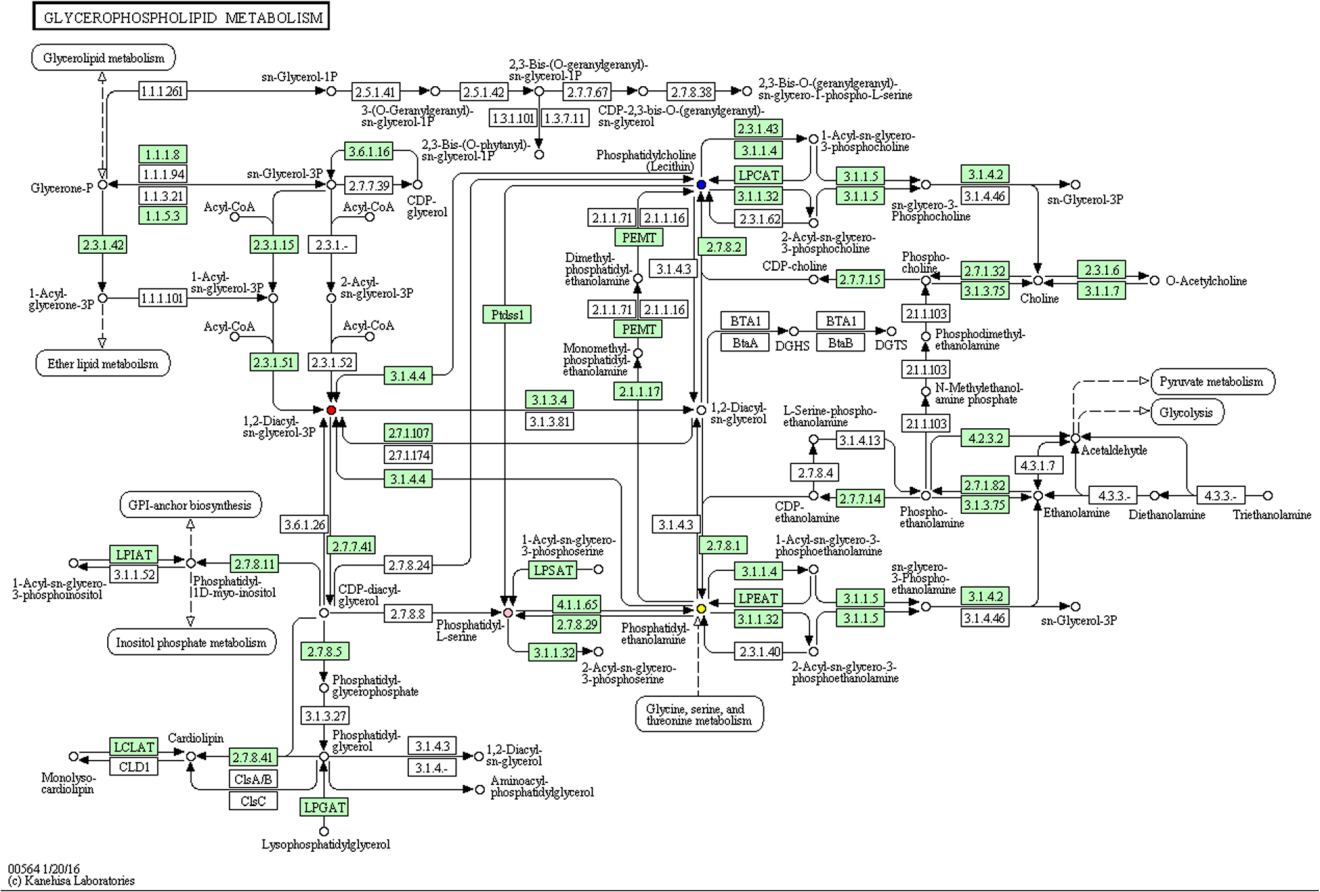
The potential mechanisms for the selected lipid species. The lipid species in the glycerophospholipid metabolic pathway were labeled with different color: PC (blue), PE (yellow), PS (pink) and PA (red)

Among these three groups, the selected lipid species were so similar to the LPEs, the subclasses of PEs. Based on the identified lipid markers, a metabolic pathway analysis was performed by KEGG pathway database, revealing the interrelationships of these selected lipid species. As shown in Fig. 5, all these selected lipid species were of great significance in the glycerophospholipid metabolism pathway. Further analyses revealed that PA was derived from PC and PE via the phospholipase D1/2 (Fig. 5). PS was derived from PC via the phosphatidylserine synthase 1. PE and PS were transmuted into each other via various enzymes.

## Discussion

A total of 114 subjects in our study were included. Although there were more Caucasian than African American among three groups, there was no significant difference in race and age (*P* > 0.05). Human plasma contains thousands of individual lipid molecular species. We could detect various lipid molecules in biological samples through liquid chromatography electrospray ionization tandem mass spectrometry (LC-ESI-MS/MS). Quehenberger et al. had reported reference values of 500 plasma lipid species which were obtained from the pooled plasma of 100 healthy people by a lipidomics analysis [21]. In our study, we had quantified 367 lipid species in each sample, and most of them were overlapped with these 500 lipid species. Due to the lack of significant difference between the BPH group and the control group, the changes of concentration of lipid species had specificity for the diagnosis of prostate cancer.

Different from many other cancer types, the prostate cancer predominantly utilizes fatty acids, rather than glucose, as energetic substrates [22]. Zaidi et al. reported that the lipids were essential for its supportive role in prostate cancer cell proliferation [23]. Interestingly, miRNA-21 and -152 did not show any expression changes in prostate cancer compared to BPH or normal control, though many miRNAs have shown altered expression levels in various adenocarcinomas of breast, lung, colorectal, and prostate cancers as well [24–27]. This fact suggests that the roles of these two miRNAs may elicit the differences in prostate cancer and other adenocarcinomas. It may be worth examining the relationship between these miRNAs and our lipids.

Since the LPEs belong to the subclasses of PEs, the selected lipid species in our study was so similar. However, our result showed that the plasma concentrations of lipid species were not the same, even in the same lipid class. The individual heterogeneity might be the major reason for the differences. After pathway analysis, we could find that these selected lipid species play a key role in the glycerophospholipid metabolism. Glycerophospholipids are the main component of biological membranes. Becoming a structural component of cell membranes for glycerophospholipid is one of its functions. PC is the major glycerophospholipid in eukaryotic cells and is an essential component in all cellular membranes [28]. It has great impact on membrane-mediated cell signaling and phosphatidylcholine transfer protein activation of other enzymes [29]. PE is significant in membrane fusion and disassembly of the contractile ring during cytokinesis in cell division [30]. PE acts as an important precursor, substrate, or donor in several biological pathways [31]. PS is a vital phospholipid membrane component which plays a key role in cell cycle signaling, specifically in relationship to apoptosis [32]. PA is the precursor for the biosynthesis of many other lipids, which acts as a signaling lipid, recruiting cytosolic proteins to appropriate membranes (e.g., sphingosine kinase 1) [33]. Therefore, we could understand why the selected lipid species could be used as diagnostic biomarkers for prostate cancer. As reported by Zhao et al., if we were to perform a plasma RNA gene expression profile and combine it with our lipid metabolic pathways, it may clarify the significance of these lipids in prostate cancer [34].

In the present study, it was observed that single lipid species was unlikely to perform well in distinguishing prostate cancer from non-malignant BPH or health individuals. However, the combination of lipid species had higher diagnostic value in prostate cancer. For the lack of plasma PSA level in our study, we were unable to compare the diagnostic efficiency of the selected lipid biomarkers with that of PSA in the same study cohort. According to the systematic calculation from American Cancer Society, the sensitivity of a PSA cutoff of 4.0 ng/mL was 21% for detecting any prostate cancer and 51% for detecting high-grade cancers (Gleason ≥8). In our study, the sensitivity and specificity for the combination of selected lipid species are all above 70%. Therefore, the combined selected lipids in our study as a panel for the diagnosis of prostate cancer was better than PSA. Fang et al. reported that the combination of the peptide hormone prolactin (PRL) with the tumor markers Carcinoembryonic antigen (CEA) and cytokeratin 19 fragment (CYFRA21) increased the diagnostic efficacy of identifying non-small cell lung cancer (NSCLC) [35]. In a similar way, it may be possible to use our lipids as a companion tool for PSA.

Apart from the sensitivity and specificity, the receiver operating characteristic (ROC) curve is used as an important index in comprehensive evaluation on the diagnosis value of a method [36]. The AUC, as the indicator for summarizing ROC, is bounded between 0.5 and 1 [37]. The AUC of the combination of selected lipid species for cancer vs. BPH group, cancer vs. control group, and cancer vs. non-cancer group was 0.800, 0.836 and 0.837, respectively. A similar result was reported by Min HK et al. [38], who found that a few phospholipids in urine were identified as potential markers for prostate cancer using shotgun lipidomics, and Zhou et al. [39], who reported that three classes of plasma phospholipids could be considered as biomarkers in diagnosis of prostate cancer by lipidomics and bioinformatics.

Our study also provided evidences that the combination of lipid species had a certain value in prostate cancer diagnosis. With the use of more bioinformatic examination, the ratio-based method proposed by Deng et al. may be an appropriate means to create novel biomarkers for prostate cancer [40].

Together with all these advantages of LC-ESI-MS/MS technology and lipidomics, this diagnostic model could be used for high-speed screening of a large number of samples for prostate cancer. These results provided a guideline to screen potential markers in diagnosis of prostate cancer.

However, this study still had some limitations. Firstly, the sample size was too small to conduct correlation analysis between the lipid species and tumor size due to the lack of related information of the prostate cancer patients. Secondly, the diagnostic value of lipid species in early-stage prostate cancer patients was not elevated. Therefore, the results still needed to be confirmed in further studies with rigorous design, larger sample size, and multiregional cooperation.

## Conclusion

This study assessed the combination of lipid species as a panel for the diagnosis of prostate cancer. These findings suggest that the combination of the identified lipid biomarkers plays an important role in the diagnosis of prostate cancer and may provide a new diagnostic strategy for prostate cancer patients.

## Materials and methods

### Patients and plasma samples collection

The plasma samples were obtained from the Rush University Medical Center during 2011–2013. The Institutional Review Board (IRB) of Rush University Medical Center approved our study. Before collecting plasma samples, written informed consents were obtained from patients and control individuals. Subjects were divided into three groups. The prostate cancer group comprised 30 patients diagnosed by subsequent prostate biopsy or prostatectomy. The prostate biopsy was performed according to the conventional method. Patients who were suspicious for having cancer underwent needle biopsy. Ten to 12 specimens were taken and processed for pathological diagnosis. The BPH group comprised 38 patients with BPH, who were also pathologically diagnosed by biopsy during the same period. Finally, the healthy control group comprised 46 male individuals who were health check-up examinees and showed no clinical manifestations of prostate diseases. The subjects with other diseases which might affect lipid metabolism such as hyperlipidemia, diabetes, and other cancers were excluded. Other clinical information for each patient was also recorded, including age, race, and pathological diagnosis, as shown in Table 1. All the patients should be fasted for 12 h before blood collection. In all the subjects, the whole blood was collected in a vacuum blood collection tube containing ethylene diamine tetra acetic acid (EDTA) (BD, Franklin Lakes, NJ) as anticoagulant. The plasma was promptly isolated after being collected and stored at − 80 °C immediately. All plasma samples were transported to the Kansas Lipidomics Research Center (KLRC) with dry ice for lipid analysis.

### LC-ESI-MS/MS lipid profiling

Triple quadruple liquid chromatography electrospray ionization tandem mass spectrometry (LC-ESI-MS/MS) (API 4000, Applied Biosystems, Foster City, CA) was used to detect lipid species profile. The details of lipid profiling were described in our previous article [41].

### Statistics analysis

SPSS 20.0 was used to analyze the data. Mean, range, and standard deviation were used for descriptive statistics. The student’s t-test was used to compare mean plasma concentrations of 367 apparent lipid species with the mean ages among the three groups of subjects. The chi-square test was used to compare the differences between the races of the African American and Caucasian. The *P* value < 0.05 was considered to be statistically significant. Scatter plots were described by GraphPad Prism Version 5 for Windows.

Weka 3.6 software was used to perform further analysis. Simple logistics classification algorithm and 10-fold cross validation were used to estimate the performance of a predictive model. The satisfactory model was used to predict the diagnostic efficiency of selected lipid species. The sensitivity, specificity, and AUC of the lipid species were calculated in accordance with the predictive model.

Two inclusion criteria for selecting the lipid species biomarkers from hundreds of lipid species were as following: (1) the *P* value was statistically significant (*P* < 0.05) and the absolute value of the fold-change was > 1.4; (2) the positive predictive value (PPV) or negative predictive value (NPV), and ROC curve were all above 0.0%.

KEGG pathway database was used to perform metabolic pathway analysis. All the selected lipid species were taken into account. The copyright permission was approved by the Kanehisa laboratory.

## Data Availability

The datasets used and analyzed during the current study are available from the corresponding authors if reasonably requested.

## Abbreviations

BPH: Benign prostatic hyperplasia
KEGG: Kyoto Encyclopedia of Genes and Genomes
AUC: Area under curve
PSA: Prostate-specific-antigen
EDTA: Ethylene diamine tetra acetic acid
PPV: Positive predictive value
NPV: Negative predictive value
ROC: Receiver operating characteristic
LC-ESI-MS/MS: liquid chromatography electrospray ionization tandem mass spectrometry
PRL: Prolactin
CEA: Carcinoembryonic antigen
CYFRA21: Cytokeratin 19 fragment
NSCLC: Non-small cell lung cancer
KLRC: The Kansas Lipidomics Research Center
